# Junction Characterization in a Functionally Graded Aluminum Part

**DOI:** 10.3390/ma12213475

**Published:** 2019-10-24

**Authors:** Elisa Fracchia, Federico Simone Gobber, Mario Rosso, Marco Actis Grande, Jana Bidulská, Róbert Bidulský

**Affiliations:** 1 Politecnico di Torino, Department of Applied Science and Technology (DISAT), Viale T. Michel 5, 15121 Alessandria, Italy; elisa.fracchia@polito.it (E.F.); federico.gobber@polito.it (F.S.G.); mario.rosso@polito.it (M.R.); marco.actis@polito.it (M.A.G.); 2 Department of Plastic Deformation and Simulation Processes, Institute of Materials and Quality Engineering, Faculty of Materials, Metallurgy and Recycling, Technical University of Kosice, Vysokoskolska 4, 04200 Kosice, Slovakia; jana.bidulska@tuke.sk; 3 Agency for the Support of Regional Development Kosice, Kosice Self–Governing Region, Strojarenská 3, 04001 Kosice, Slovakia

**Keywords:** aluminum FGM, impact test, EN AC 42100, EN AC 48000

## Abstract

Aluminum alloys are widely used to produce automotive components, thanks to their great mechanical properties–to–density ratio. Engine components such as pistons are conventionally produced by casting of Al–Si eutectic alloys (Silumin alloys) such as EN AC 48000. Due to the harsh working conditions and the lower ductility if compared to aluminum–silicon alloys with lower silicon content, pistons made of this alloy are prone to fatigue failures in the skirt region. In order to overcome such limits, the use of a Functionally Graded Material (FGM) in the production of a piston is proposed. The adoption of a functionally graded architecture can maximize the properties of the component in specific areas. A higher level of thermal resistance in the crown of the piston can be achieved with EN AC 48000 (AlSi12CuNiMg), while higher elongation at rupture in the skirt region would be conferred by an EN AC 42100 (AlSi9Mg0.3). The FGM properties are strictly related to the metallurgical bonding between the alloys as well as to the presence of intermetallic phases in the alloys junction. In the present article, the characterization of gravity casted FGM samples based on Al–Si alloys with respect to microstructure and mechanical testing is presented, with a specific focus on the characterization by impact testing of the joint between the two alloys.

## 1. Introduction

Functionally Graded Materials (FGMs) are advanced composite materials characterized by variations in composition and/or microstructure along the entire piece, with the aim to obtain specific properties in different areas of the part [1,2]. The gradual variation of composition and/or microstructure along the FGM volume permit differentiated responses to an applied solicitation [3,4]. The microstructural variation in the volume of metallic FGM is due to the presence of two or more phases metallurgical bonded to each other. In this sense, the transition between the two phases can be critical for the presence of a junction that has to be continuous, ensuring a gradual modification of both composition and properties into the entire volume, minimizing the severe risk of introducing local defects.

FGM parts may be produced by different methods such as gas–based methods, liquid–phase methods and solid–phase methods, while metallic FGMs are commonly obtained by casting techniques (centrifugal casting, squeeze casting, gravity casting) [1,2,3,4,5].

Several fields of application are relevant for FGMs: automotive, aerospace, biomedical and energy sectors [6,7,8,9]. Recent FGM research [10] has been focused on several classes of materials and production processes such as chemical vapor deposition, physical vapor deposition, additive manufacturing and casting methods [11]. For example, FGMs in SiC–Al alloy can be used for Diesel engine piston and brakes, while FGMs made of epoxy resin–glass are used for sonar domes in sub–marine applications [3]. The use of aluminum alloys is particularly common for the automotive sector. In recent years the aluminum alloys market expansion has been guided by aerospace and automotive industries, because of the stricter environmental demands [12,13,14,15,16,17]. Particularly, car components made of casted aluminum alloys are common features in everyday cars (piston, engine blocks, rocker coves [18]); permanent mould casting processes are still leading the production market of structural components. Furthermore, aluminum alloys are sustainable class of materials, recyclable [19] with cost–effective processes and characterized by good mechanical resistance, lightweight and weldability [20,21,22].

Recent works have suggested the production of aluminum–FGM pistons by gravity casting [23,24,25,26] adopting a dual, functionally graded, composition: EN AC 48000 (AlSi12CuNiMg) in the piston crown ensuring mechanical strength and thermal resistance, and EN AC 42100 (AlSi7Mg0.3) in the piston skirt providing higher ductility. In fact, pistons are commonly made of Silumin alloy EN AC 48000 and are often subjected to fatigue cracking in the skirt because of the low alloy ductility [27,28,29]. Furthermore, in these alloys, the presence of Cu and Mg, either present in intermetallic phases or into the α–solid solution, can affect corrosion resistance. Particularly, Zeng et al. [30] demonstrated that Mg_2_Si intermetallic and Si particles influenced the aluminium–silicon alloys corrosion rate. In corrosive environment, Mg_2_Si has initially an anodic behaviour while silicon has a cathodic behaviour and corrosion may occurs on Mg_2_Si surface. During Mg_2_Si corrosion process, the preferential dissolution of Mg causes an enrichment of Si in Mg_2_Si intermetallic leading to the anodic dissolution and corrosion of the base alloy. As Cu regards, Al_2_Cu create a galvanic couple with aluminum matrix: Al_2_Cu act as a cathode accelerating the oxidation of the α–Al phase [31].

Since aluminum–silicon alloys are widely adopted in automotive applications, their mechanical properties are well known [23,24,25]; conversely the mechanical properties of the aluminum–FGM are not widely known. Indeed, the joint between the alloys can represent a weak point for low metallurgical bonding due to shrinkage, entrapped air and oxides and bifilm that are intrinsically generated by the casting process [32,33,34,35,36] which may affect the mechanical resistance of the FGM.

Since the production standards in the automotive sector require high quality castings and large series production, it has to be proved that the junction between the alloys in the FGM does not represent a preferential site of defectiveness and failure. For this reason, the characteristics of the aluminum–aluminum FGM obtained via gravity casting have been investigated in this work. FGMs were obtained as previously seen in [25] and T6 heat treated [37]. The joint between the two alloys, is a metallurgical bonding between them, for this reason this sort of metallurgical interface was studied by means of micro–hardness tests, impact tests, and SEM microstructural analysis, in order to characterize the quality of the bonding between the two alloys and investigate the intermetallic nucleation near the alloys junction. Both the mechanical characterization and the intermetallic observation approach were useful in emphasizing any possible critical issue.

## 2. Materials and Methods

In this work, two aluminum alloys belonging to the Silumin group have been used. EN AC 42100 (hereinafter called alloy 1) belongs to the hypoeutectic system Al–Si–Mg (see Table 1 for composition) and is characterized by a low alloying element content and high ductility. The hardness of EN AC 42100 alloy may be increased by T6 heat treatment, thus achieving strengthening by the precipitation of the intermetallic Mg_2_Si. EN AC 48000 (hereinafter called alloy 2) has an eutectic composition and belongs to the Al–Si–Cu–Ni–Mg system (its composition is reported in Table 1) containing alloying elements as Ni, Cu and Mg that influence respectively the thermal fatigue resistance [29,38] and the mechanical resistance: Cu already in as–cast conditions, Mg after heat treatment [39]. This specific alloy composition is often called piston alloy because it is commonly used to cast these parts and is characterized by high mechanical resistance and low ductility [40]. Its mechanical resistance can be improved by heat treatment T6 promoting the nucleation of strengthening intermetallics as Mg_2_Si, Al_2_Cu, Al_3_Ni and other more complex intermetallic phases [41].

FGMs casting procedure has been carried by manual casting the two alloys in a pre–heated steel mould. The mould surfaces were prepared by sandblasting and painted with a BN–based stop off paint to prevent casting from sticking during ejection [42,43]. No grain refinement or modification of The alloys were casted as it, without any addition of grain refiners or modifiers.

Melting of the alloy was performed in an electric furnace with a graphite crucible; the gravity casting process was operated manually and was guided by some key–parameters [25] that play an essential role in the production of an FGM with good interface quality. These parameters are the casting sequence, the soaking temperatures of the two alloys, the mould pre heating temperature and the elapsed time between the casting of the two alloys. Considering the nature of the casting performed (manual mode), temperatures higher than liquidus have been chosen: 710 °C for alloy 1 and 750 °C for alloy 2. The mould was thermalized to 400 °C in order to avoid thermal shock during casting and have a proper solidification of the casting.

**Figure 1:**
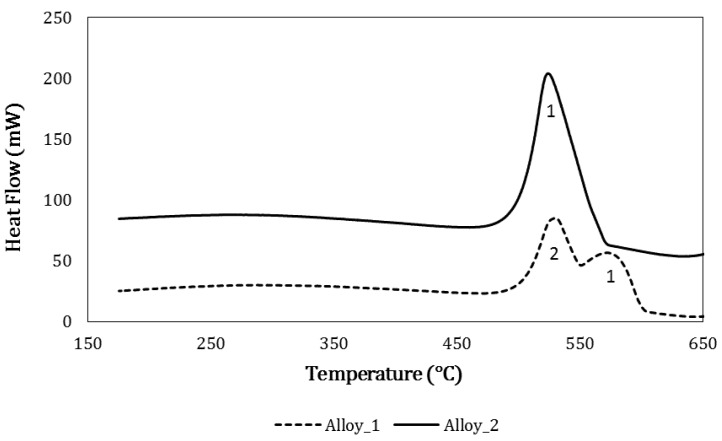
Differential Scanning Calorimetry (DSC) graph obtained for both the alloys during cooling with Seratam TGA–DSC9216.18.

Casting sequence depends on the solidification interval, influencing the metallurgical bond between the alloys. In particular, alloy 1 has a large solidification range (Figure 1) while alloy 2 has a minor gap. In this sense, in Figure 1 the Differential Scanning Calorimetry (DSC) thermograph during cooling form liquid is reported for both alloys (TGA–DSC9216.18, Seratam, Caluire-et-Cuire, France).

For alloy 1 the thermal events shown in Figure 1 are [44]:

(1) Peak 1=Liquid→α(Al)+Si+(other alloying elements)

(2) Peak 2=Eutectic solidification

For alloy 2 [45,46]:

(3) Peak 1=Liquid→α(Al)+Si+(other alloying elements)

The wider solidification interval of alloy 1 allows one to obtain a barrier effect when pouring alloy 2, thanks to the primary solidification of the *α*–aluminum–phase that avoids the two alloys from mixing completely. In the area of contact between alloy 1 and alloy 2, the partial remelting of alloy 1 generates a gradient microstructure. The metallurgical bonding is stabilized by alloy 2 infiltrating into the interdendritic channels of alloy 1. This production process permits to obtain FGMs with a very thin interface that can give important information about junction properties that can be transferred to larger parts. The pouring interval influences the bonding between the two alloys and the defects formation (bifilm, entrapped bubbles) [47]. In a previous study [25], it was argued that defects at the interface between the alloys may be reduced by introducing a 20 s pouring interval; castings were then subjected to heat treatment T6. According to literature [37,48,49,50], castings were solution heat treated at 530 °C for 8 h and then water quenched at 25 °C and aged at 175 °C for 4 h [26].

Casting obtained are square bars of 25 mm × 125 mm × 15 mm with the alloys–junction located approximately in the middle of the bars. The size of the casted parts was chosen as the one that can permit the production of different kind of samples (tensile specimens, impact specimens, fatigue specimens). After casting, samples were analyzed in terms of microstructure and mechanical properties, focusing on the properties of the metallurgical junction between the two alloys. After each test, specimens were analyzed by SEM analysis (SEM LEO 1450VP, Carl Zeiss Microscopy GmbH, Jena, Germany, equipped with an Oxford Link Pentafet probe, INCA Energy, Oxford, UK).

### 2.1. Microstructural Characterization

The FGM sample for microstructural analysis was cut from a casted ingot, heat treated T6 and polished using SiC papers from 180 grit up to 2400 grit than polished on a cloth with colloidal silica and finally etched with Keller’s reagent for 40 s. Microstructural analysis focused on: (I) alloys microstructures; (II) alloys interface; (III) intermetallic phases at the interface, (IV) eutectic silicon particles size. After the microstructural observation (optical microscope LEICA MEF4M, Leica Microsystems, Heerbrugg, Switzerland) the same specimen was used to perform the micro–hardness indentations. By means of image analysis software (LEICA QWin, version 3.5, Leica Microsystems, Heerbrugg, Switzerland), the eutectic silicon average size in bulk and near the interface was measured for each alloy on a polished sample.

### 2.2. Micro–Hardness

Micro–hardness measurements were performed along the FGM interface by a microhardness tester with parameters 15 s, 50 Kgf–HV 0.5 (LEICA VMHT, Leica Microsystems, Heerbrugg, Switzerland). Measurements were made along parallel lines crossing the interface to detect variation in local hardness. A 100 µm step was adopted between one indentation and the subsequent one. The micro–hardness test was chosen instead of macro–hardness because it can give important information about the intermetallic phases nucleated at the interface between the alloys. In fact, microstructural elements (intermetallic as well as silicon particles) are larger than the actual indentation: this can permit to recognize the difference between the soft matrix hardness in alloy 1 (alloy with minor alloying elements), the hard phases (intermetallics) and the soft matrix in the alloy 2 (alloy rich of alloying elements). Moreover, with a dense network of indentations may be possible to detect intermetallic phases typical of alloy 2 nucleate into alloy 1, as a demonstration of metallurgical bonding between the alloys.

### 2.3. Impact Test

**Figure 2:**
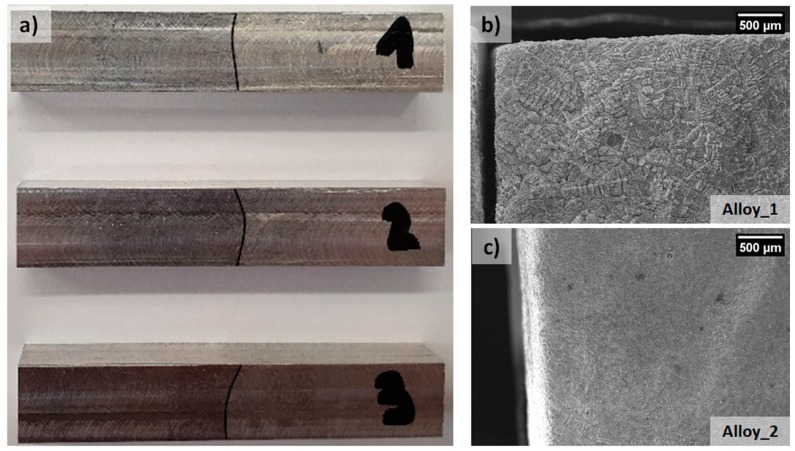
(**a**) Macrograph of the samples for impact testing. The interface was highlighted with a marker. On the right side (images **b** and **c**) deep–etched fracture surface evidenced the alloy EN AC 42100 (**b**), characterized by large dendritic structures, and the alloy EN AC 48000 (**c**), with finer dendrites.

Samples required for impact tests are prepared, after T6 heat treatment, following the ASTM E23 standard (55 mm × 10 mm × 10 mm); the junction between the two alloys as left in the central position after machining of samples. Due to the non perfect linearity of the joints (see Figure 2) and to the low amount of kinematic energy expected to be absorbed [51,52], samples were not pre–notched. For impact testing a TG5113E instrument has been employed (Zwick Roell, Genova, Italia). One of the two halves of each fractured sample was investigated by means of SEM analysis while the other half was deep etched with pure HCl (35% vol.). By deep etching with HCl, a selective corrosion process acts on α–aluminum exposing the sole eutectic and intermetallic microstructures: by employing this method it is possible to determine the fracture path at the interface between the two alloys (see Figure 2b,c). After deep etching the samples, the relative percentage of each alloy on the fracture surface was quantified by image analysis.

## 3. Results and Discussion

### 3.1. Microstructural Characterization

When heat treating the as–casted FGM several dissolution and precipitation processes take place. After solution heat treatment, the alloy is a supersaturated solid solution (SSS). During the whole heat treatment, the thermal energy leads to the propagation of competitive phase transformation mechanisms such as dissolution of intermetallic and spheroidization of the needle shaped eutectic silicon by progressive fragmentation and rounding [53,54]. The presence of magnesium in both alloys leads to the formation of the β–Mg_2_Si. Additionally, when iron is present, both the alloys tend to form different intermetallic compounds such as the α–Al_8_Fe_2_Si, β–Al_5_FeSi, π–Al_8_Mg_3_FeSi_6_ and Al_9_FeMg_3_Si_5_ [55,56].

Despite the poor solubility of iron in aluminum, Fe–based intermetallic dissolution may be possible by solution heat treating at higher temperature [55]: β–Al_5_FeSi may change its morphology by fragmentation and dissolution while the α–Al_15_(Mn,Fe)_3_Si_2_ does not change its shape [57]. In alloy 2 the presence of both copper and magnesium leads to the formation of the θ–Al_2_Cu, Al_2_CuMg and Q–Al_5_Cu_2_Mg_8_Si_6_ while the presence of Mn leads to the formation of the Chinese–script like α–Al_15_(Mn,Fe)_3_Si_2_ intermetallic [58]. Alloy 2 (AlSi12CuNiMg) could additionally form the phases: ε–Al_3_Ni, γ–Al_7_Cu_4_Ni, δ–Al_3_CuNi(Al_3_Ni_2_), T–Al_9_FeNi [41] and α–Al_11_(MnFeNiCu)_4_Si [58]. In [59] it is suggested that some intermetallic play an active role in the resistance to corrosion: intermetallic particles such as the Al_2_CuMg and Mg_2_Si show anodic behaviour acting as preferential corrosion site while intermetallic phases such as Al_2_Cu, AlFeMnSi, AlCuFeMn, AlCuFeSi, show cathodic behaviour causing the corrosion of the surrounding α–Al matrix. Because of the contemporary presence of several different intermetallic phases, the eutectic alloy (alloy 2) is less corrosion resistant than the hypoeutectic alloy (alloy 1). In this sense, the junction between the alloys in corrosive environment may be affected by a corrosive process led by the alloy 2 penetration into the interdendritic channels of alloy 1.

**Figure 3:**
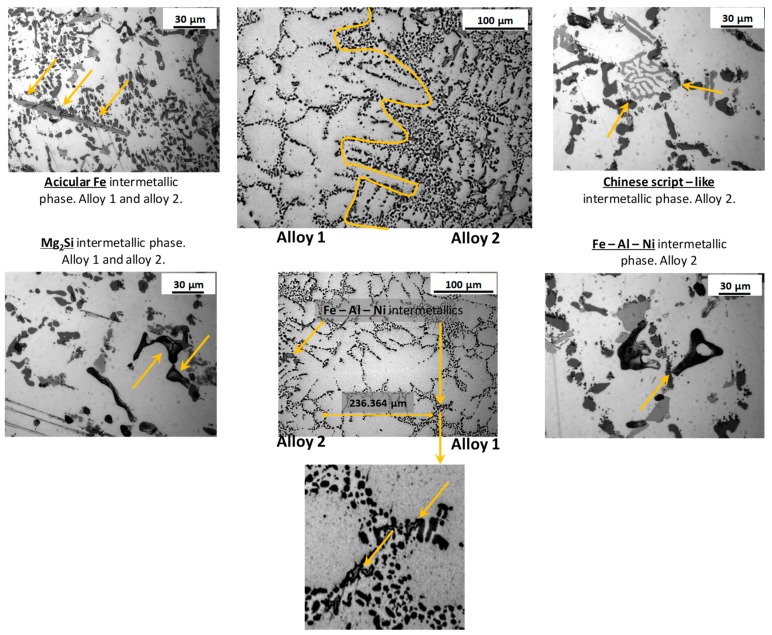
Light optic micrograph of the intermetallic phases in the two alloys and FGM microstructure. The acicular Fe– phase, the Mg_2_Si intermetallic and the Chinese script–like intermetallic are present in both the alloys while Fe–Ni–Al intermetallic phase is present only in alloy 2 and near the alloys junction. The interface between the alloys is highlighted in the central microstructures in the upper by the solid orange curve. In the lower central micrographs are showed the intermetallic phases Fe–Al–Ni, typically present in alloy 2, into the interdendritic channels of alloy 1.

**Figure 4:**
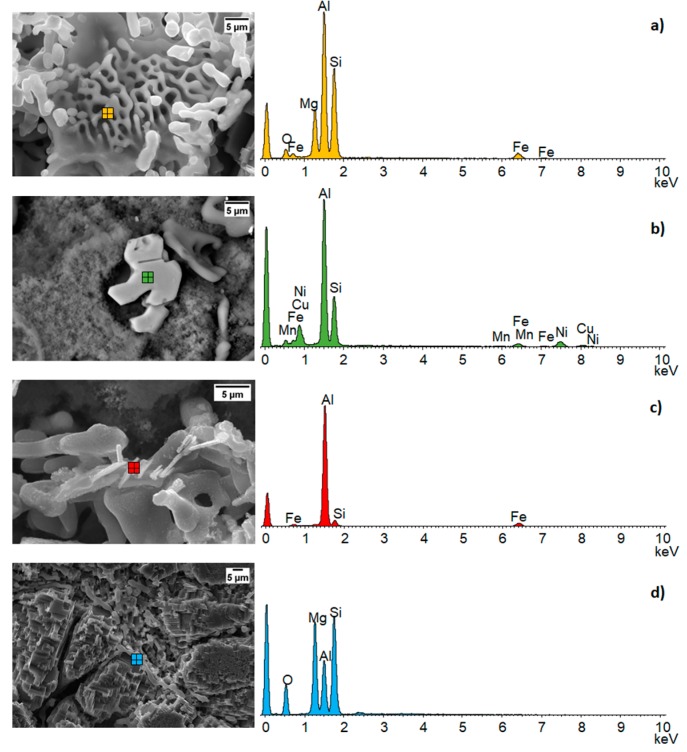
SEM micrographs and EDS analysis of the intermetallic phases in the two alloys and previously shown in Figure 3. (**a**) Chinese script like; (**b**) Fe–Al–Ni based intermetallic; (**c**) Al–Fe–Si based acicular intermetallic; (**d**) Mg2Si intermetallic.

The T6 heat treatment acts with a time/temperature dependent effect on the spheroidization of eutectic silicon too [54]. Particularly, silicon spheroidization process is important because the acicular shaped silicon acts as a preferential site for fragile fracture propagation. In this sense, the difference in the degree of silicon spheroidization at the interface between the two alloys may have an effect on the interfacial resistance to impact. Both the microstructure at the interface of the FGM and some common intermetallic phases are shown in Figure 3 and Figure 4. In Figure 3 intermetallic phases Fe–Al–Ni, typically present into alloy 2, were detected into the eutectic phase of alloy 1 at 236 µm of distance from the interface with alloy 2.

In Figure 4 are shown SEM micrographs and the EDS analysis for the main intermetallic phases present into the FGM microstructure. These intermetallics influence both the mechanical properties, in terms of hardness, and the impact properties, affecting the amount of energy absorbed. Rounded shapes increase the energy absorption promoting the ductile fracture while polygonal shapes promoting the fragile fracture reducing the energy absorption.

### 3.2. Micro–Hardness

Micro–hardness measurements have been carried out on a polished FGM specimen with a surface of 20 mm × 20 mm, along the interface between the two alloys (total area involved in indentations 2.5 mm × 0.3 mm). The infiltration of alloy 2 into the interdendritic channels of alloy 1 leads to an increase in hardness of alloy 1 thanks to the nucleation of Ni, Fe and Cu–based intermetallic.

**Figure 5:**
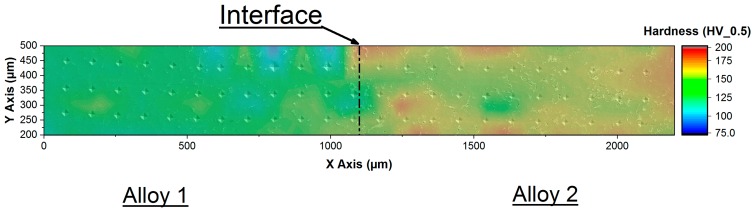
Micro–hardness map. Vickers indentations along three lines crossing the interface between the two alloys. The coloured map on the image identifies the hardness values detected, as shown in the legend on the right side of the image.

Figure 5 shows the micro–hardness profiles for the FGM sample. As attended, the maximum and minimum hardness values were found respectively in alloy 2 (190.4 HV0.5) and in alloy 1 (77.1 HV0.5). Abrupt variations in hardness values were due to the kind of intermetallic indented [60]. In alloy 1, the nucleation near the interface of hard complex intermetallics causes an increase in hardness values. The SEM micrograph in Figure 5 shows details of the indented phases: hardness marks appear small in correspondence of hard intermetallic and large in correspondence of α–dendrites.

### 3.3. Impact Test and Fracture Analysis

**Figure 6:**
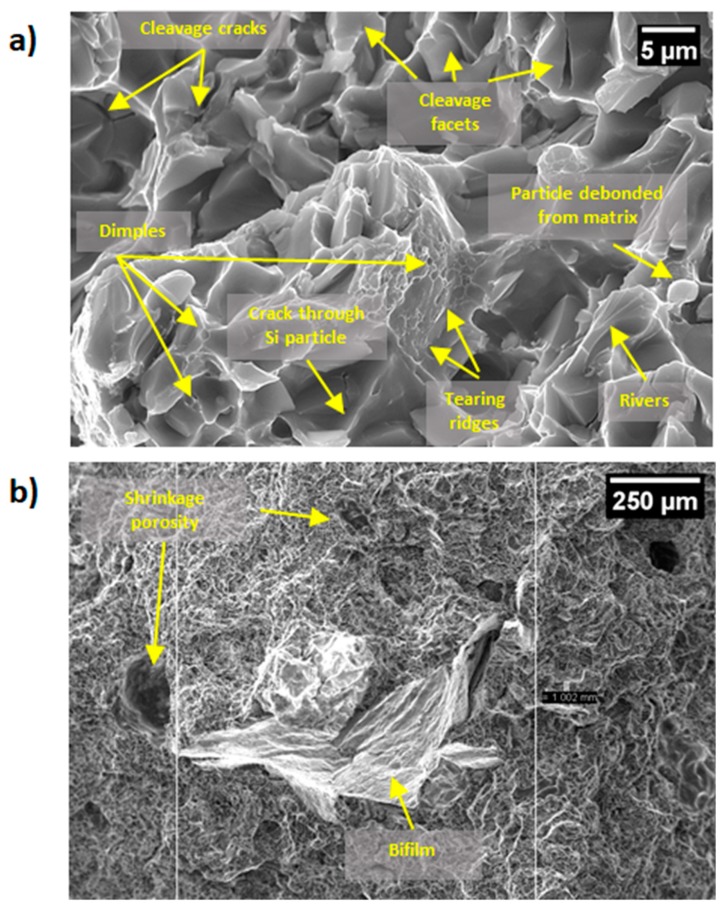
SEM micrographs of the fracture surface for an impact test specimen at higher magnification (**a**) and at lower magnification (**b**). In the micrographs could be observed cleavage plains, shrinkage porosities, bifilm and dimples.

From impact tests performed on non–notched FGM samples an amount of energy two times larger than the state of the art for alloy 1 was absorbed by the specimens [52]. Cleavage plains, some shrinkage porosities and dimples are detectable from the observation of the fracture surfaces of the tested samples (see Figure 6). In [61] it was demonstrated that ductile fractures occur by plastic strain around second phase particles such as eutectic–silicon particles, causing Si–particles debonding and cracking; this mechanism is noticeable in Figure 6 too. Fracture propagation paths influenced the absorbed energies. After the analysis of the fracture surface it has been evidenced that if the fracture mainly involves large oxide skins or large shrinkage cavities, the absorbed energy decreases, resulting in a predominantly brittle fracture mode. The heat treatment T6 must properly modify silicon from acicular to spherical shape, in order to avoid fragile fractures [62,63]. Alloy 2–T6 microstructure is characterized by brittle Fe–Mn–Ni–based intermetallic and Mg_2_Si intermetallic, spheroidized silicon particles and Al_2_Cu nano–particles leading to a transcrystalline–ductile fracture mechanism. The presence of fine Si–particles resulted in a significant improvement in the fracture toughness owing to their influence on the void nucleation process while micro–voids nucleated and grew from dispersion strengthened phases as θ and θ’ (Al_2_Cu). Alloy 1 microstructure is characterized by an α–aluminum matrix with embedded spheroidized silicon particles and β–phase (Mg_2_Si): eutectic silicon particles are the only void nucleation initiators in this alloy.

Both heat treatment and the type of casting influence the size of the silicon particles. In this case, in alloy 1 the measured silicon average size was 6.821 µm^2^ in the bulk while it was 11.108 µm^2^ close to the junction. In alloy 2 silicon average size was 17.741 µm^2^ in the bulk and 5.585 µm^2^ close to the junction. The change in silicon size from bulk to junction may be explained by the fact that during casting, the infiltration of alloy 2 into the interdendritic channels of alloy 1 provides additional heating causing an increase in the eutectic silicon size of alloy 1. On the other hand, when pouring alloy 2, alloy 1 is partially solidified causing a quick cooling of the alloy 2 and a reduction in the silicon average size. Since silicon average size influenced dimples formation, the surface fracture may be related to silicon average size measured near the interface.

Dimples theoretical average size in the FGM interfaces was calculated based on the amount of alloys involved in the fracture and on the silicon average size obtained by image analysis:

(4) $$Dimples_{theoretical\;size}=Si_{avg\;size\;alloy\;1}\%\;alloy\;1+Si_{avg\;size\;alloy\;2}\%\;alloy\;2$$

**Figure 7:**
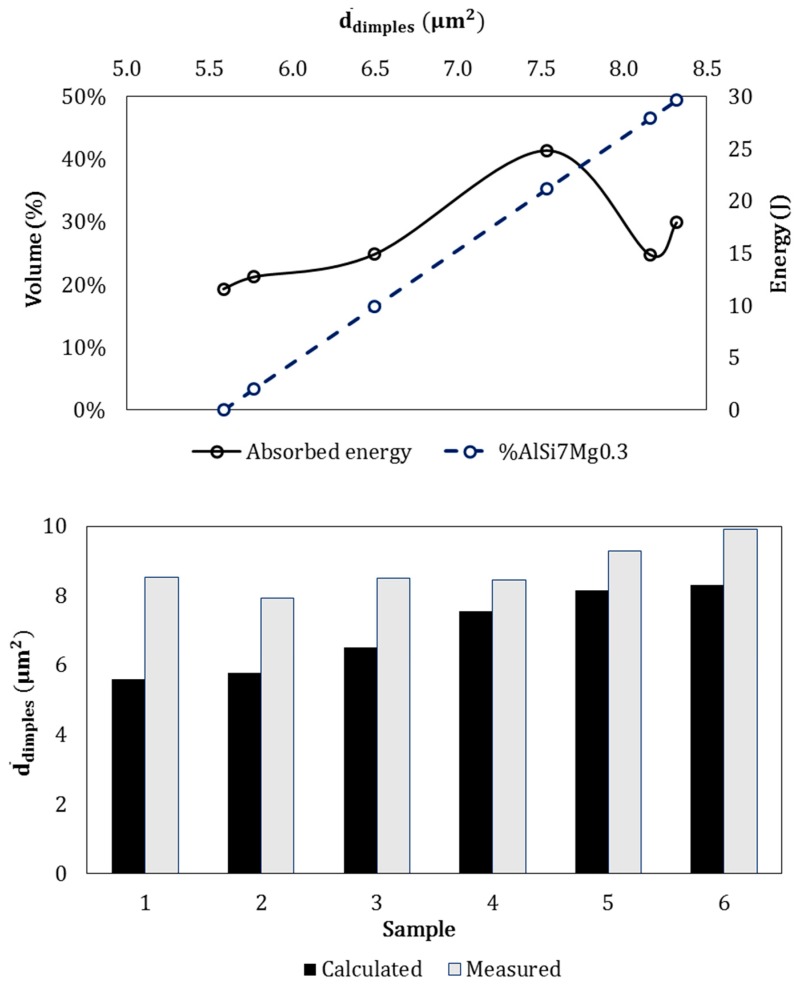
(**Top**): graphical relation Energy absorbed–dimples average size calculated by image analysis The graph also shows the correlation Energy absorbed–% of alloy 1 detected in the fracture surface. (**Down**): difference between dimples average size measured on surface fracture and calculated by image analysis.

Alloy 2 casted in the FGM, is characterized by a non–negligible level of porosity and a relevant amount of fragile intermetallics, thus when the fracture involved high amount of alloy 2, the absorbed energy decreased if compared to other samples with a higher fraction of alloy 1 at the interface. Moreover, the nucleation of brittle intermetallic compounds in alloy 1 near the junction influenced the dimples real size causing deviations from the theoretical values (see histograms in Figure 7 Down). In the overall, an increase in dimples average size with the increase of quantity of alloy 1 was observed (dotted line in Figure 7 Top). Moreover, the measured dimples size differ to the theoretical ones. This was probably due to: (I) difference between silicon average size calculated by image analysis and silicon average size in the specific sample; (II) the dimples theoretical size was calculated assuming the dimples size identical to the silicon size; (III) relevant amount of porosities in alloy 2 influenced the fracture behavior, especially with higher amounts of alloy in the fracture surface. Speculating on absorbed energy, there is a discernible trend in the increase of dimples average size with the increase in absorbed energy and percentage of alloy 1, as attended, due to its most ductile composition. Furthermore, in Figure 7 lower energies correspond to a higher average size of dimples. This behavior is due to the presence of defects on the specimens interface such as bifilm and porosities causing a reduction of the absorbed energies (see Figure 6). Such defect are intrinsically part of the casting process of aluminum alloys [64].

## 4. Conclusions

In this work, the junctions of aluminum alloy based–metallic functionally graded materials produced by sequential gravity casting were characterized, from the point of view of mechanical properties and fracture resistance. After sequential gravity casting, metallurgically bonded joints between the two alloys are solid with continuous interface. Far from the metallurgical junction, the two microstructures appear completely different one from the other in accordance with the microstructures of each of the two alloys. In the surroundings of the metallurgical junction the penetration of alloy 2 into the eutectic channels of alloy 1 led to the formation of the metallurgical bonding and to the nucleation of Fe–Ni based intermetallic into the hypoeutectic alloy (alloy 1), causing its increase in hardness. Impact tests highlighted the effect of the junction between the two alloys. In fact, the presence of defects, brittle intermetallic and porosities negatively affect the impact energy while the presence of high amount of alloy 1 on the fracture surface lead to higher impact energies. Moreover, a relation is proposed between the theoretical silicon average sizes measured by image analysis (µm^2^) and the amount of alloy involved into the fracture. Good accordance was highlighted between the dimples measured experimentally and the trend predicted by the relation; the higher values in dimple size measured depend on the plastic deformation of the material during the dimples formation. Normally, an impact test requires a well–defined interface in composite materials, while in this specific case the interface shape varies quite a lot. This was caused by the different bonding between the alloys during each casting. The interface shape is closely related to the casting process that, in turn, is closely related to a high number of variables. In this sense, the graph in Figure 7 clearly indicates that the interface shape may affect the absorbed energy encouraging the fragile fracture in the presence of a higher content of alloy 2. The interface in the FGM corresponds to a high metallurgical bonding, as highlighted by the diffusion of Ni–Cu and Fe–Al–Ni intermetallic phases into the alloy 1 (as showed Figure 3) and by the hardness measures.

## Figures and Tables

**Table 1:** Chemical compositions and mechanical properties of the alloys [25].

**Alloy 1—EN AC 42100—AlSi_7_Mg_0.3_**										
Elements	Si	Fe	Cu	Mn		Mg	Zn	Ti	Al	
Min (%)	6.5	–	–	–		0.25	–	–	Bal.	
Max (%)	7.5	0.19	0.05	0.10		0.45	0.07	0.25		
Tensile properties in T6 state					**Rm** 290–340 (Mpa) **A** 4%–9% **Rp_0,2_** 220–280 (Mpa)					
**Alloy 2—EN AC 48000—AlSi_12_CuNiMg**										
Elements	Si	Fe	Cu	Mn		Mg	Ni	Zn	Ti	Al
Min (%)	10.5	–	0.8	–		0.8	0.7	–	–	Bal.
Max (%)	13.5	0.7	1.5	0.35		1.5	1.3	0.35	0.25	
Tensile properties in T6 state					**Rm** 350–400 (Mpa) **A** 0.5%–2% **Rp_0,2_** 320–390 (Mpa)					
